# Terahertz electromagnetically-induced transparency of self-complementary meta-molecules on Croatian checkerboard

**DOI:** 10.1038/s41598-019-42038-8

**Published:** 2019-04-17

**Authors:** Zhenyu Zhao, Xiaobo Zheng, Wei Peng, Jianbing Zhang, Hongwei Zhao, Wangzhou Shi

**Affiliations:** 1 Department of Physics, Shanghai Normal University, Shanghai, 200234 China; 2 State Key Laboratory of Functional Materials for Informatics, Shanghai Institute of Microsystems and Information Technology, Chinese Academy of Sciences, Shanghai, 200050 China; 3 Key Laboratory of Interface Physics, Shanghai Institute of Applied Physics, Chinese Academy of Sciences, Shanghai, 201800 China

**Keywords:** Metamaterials, Terahertz optics

## Abstract

A terahertz (THz) electromagnetically-induced transparency (EIT) phenomenon is observed from two types of self-complementary meta-molecules (MMs) based on rectangular shaped electric split-ring resonators (eSRR) on Croatian checkerboard. Each MM contains a couple of identical size eSRRs and a couple of structural inversed eSRRs twisted π/2 in checkerboard pattern. In the first type of MM (type-I), the gap is in the middle line of eSRR. In the second type of MM (type-II), the gap is on the two arms of eSRR. Both types of MMs exhibit EIT effect. A maximum 20 ps group delay is observed at the transparency window of 0.63 THz in type-I MM; while a maximum 6.0 ps group delay is observed at the transparent window of 0.60 THz in type-II MM. The distribution of surface currents and electrical energy reveals that only CeSRR contribute to the transparency window as well as the side-modes in type-I MM, where the current leakage via contact point contributes to the low-frequency side-mode, and the coupled local inductive-capacitive (LC) oscillation in CeSRRs contributes to the high-frequency side-mode. In type-II MM, however, the localized dipolar oscillator of CeSRR contributes to the low-frequency side-mode; while the hybridization of dipole oscillation on eSRR and LC resonance on CeSRR contributes to the high-frequency side-modes. Our experimental findings manifest a new approach to develop THz slow-light devices.

## Introduction

Maxwell equations indicate that the electric field (E) is the dual of the magnetic field (H). As such, the diffraction energy from an opaque object is identical to that from a hole of the same size and shape to be measured, which is termed as Babinet’s principle^1–4^. According to this principle, the transmission coefficient tc for the inverse structures of meta-molecules (MMs) is related to the transmission coefficient to for the metal structural MM by to + tc = 1^1,2^. Here, the inverse structures where the metallic patterns are replaced with open areas and open areas are replaced with metal, which is termed as complementary structures. At resonance frequency, it is found that the to of metal structural MM achieve minimum, while the tc of structure inversed or complementary MM achieves maximum. Owing to this phenomenon, the metal resonators and its complementary resonators can be integrated in one unit cell, which is termed as self-complementary MMs. There are many works on the extra-ordinary response of manipulating electromagnetic wave using self-complementary MMs, such as negative permittivity and permeability^3^, frequency-independent response^4^, asymmetric transmission^5^, polarization conversion^6,7^, flat band-filter^8^, invisible cloaking^9^ controllable Smith-Purcell effect^10^, and travelling wave manipulation in accelerator^11^.

**Figure 1:**
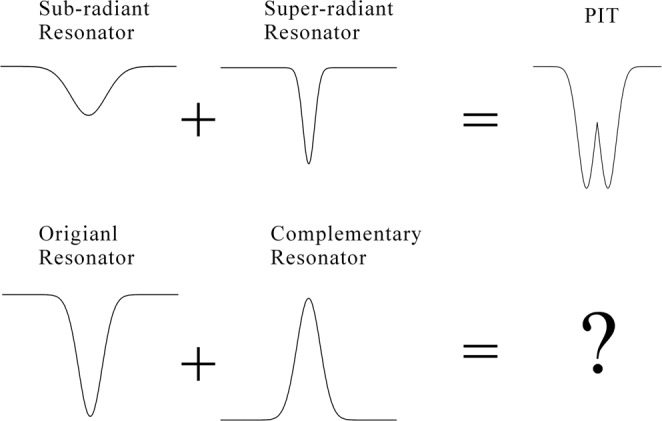
Diagram of interaction of resonators, upper-line: origin of EIT, lower-line: the interaction of metal structure resonator and its self-complementary counterpart inside MM. The black curves refer to the spectral profiles of corresponding resonators. “+” refers to the modes coupling each other.

Recently, an electromagnetically-induced transparency (EIT)-like effect is for the first time observed in a self-complementary MM combining cut-wire resonators and its pseudo-complementary pattern together, which is naturally an interference of several conductively coupled metal cut-wire resonators^12^. Actually, the EIT effect appears to be a medium transparent within a narrow spectral range around an absorption line^13–30^. The dispersion at the transparency window is extremely large so as to reduce dramatically the group velocity of the incident light. Such a slow light effect could be used to greatly reduce noise for all types of information to be transmitted more efficiently in telecommunication from optical region to terahertz band^16–21^. Normally, the EIT effect originates from a destructive interference of surface plasmon oscillation in between two coupled metal resonators having relatively close oscillation amplitude at the same resonance frequency^13–30^. Furthermore, one resonator has a relatively high Q factor mode to_H_ termed as super-radiant resonator; while the other has a relatively low Q factor mode to_L_ termed as sub-radiant resonator. Both modes achieve transmission minimum at the same THz frequency. Then, a transparency window occurs at aforementioned frequency. The principle of EIT effect is illustrated in Fig. 1. Owing to the Babinet’s principle, the central frequency of resonance minimum of the originally metal resonator to must be identical to that of the resonance maximum of the corresponding inversed structures tc. If a resonator coupled with its own inversed structure inside the same MM, whether an EIT effect will occurs or there will be totally different phenomenon?

In order to answer above question, we propose a novel self-complementary MM based on Croatian checkerboard. Each unit cell contains dual metal resonators and their inversed structure resonators. In our work, a couple of electric SRR (eSRR) works as originally metal resonators, while another couple of its complement (CeSRR) works as inversed structure resonators. The resonant frequency of eSRR is close to the CeSRR, while the Q factor of eSRR is relatively smaller than CeSRR. As such, it satisfies the requirement of occurrence of EIT effect. Compared to the bianisotropic single or double SRR, the eSRR and its inversed structure CeSRR are purely of electric response neither magnetic nor magnetoelectric response^31,32^. Thus, it can be excluded that the contribution from the net magnetic dipole moment to the interference behavior of different intrinsic modes inside the self-complementary MM. The frequency response of MM is characterized by the THz time-domain spectroscopy (THz-TDS). The origin of the transparency windows as well as the slow light effect is revealed by numerically mapping the electromagnetic field and the surface current at the frequencies of transparency windows as well as the side-modes.

## Methods

### Fabrication

The resonators of MMs are partterned on a piece of 625 μm-thick semi-insulating gallium arsenide (SI-GaAs) wafer by photolithography. The metal layer of resonators contains 120 nm-thick gold (Au) and 5 nm-thick titanium (Ti), which are deposited on the SI-GaAs wafer by vacuum evaporation. Here, the Ti is adhesion layer in between Au and SI-GaAs wafer. Each piece of as-fabricated MM is cut into rectangular section as 12.7 mm in length and 12.7 mm in width, in which the effective area of MMs is 10.0 mm × 10.0 mm. Therefore, one piece of sample contains 10000 unit cells of MM. The contact point is determined by the spatial resolution of mask aligner (Karl Süss MA6), which is less than 1 μm. Owing to the limited resolution of mask as well as the yield ratio of micro-fabrication process, the contact ratio in between the metal square of CC on the as-fabricated MM are different for the two types of MM. For type-I MM, the contact ratio is 90%. For type-II MM, the contact ratio is 84%. Above ratio are recorded from the microscopic images of type types of as-fabricated MM.

### Characterization

**Figure 2:**
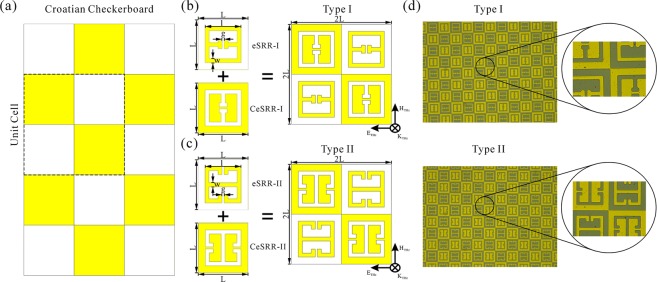
(**a**) Croatian checkerboard (CC), the dash-line enclosed area refers to one unit cell of CC. (**b**) Diagram of type-I eSRR/CeSRR and corresponding self-complementary metamolecule, (**c**) Diagram of type-II eSRR/CeSRR and corresponding self-complementary metamolecule. K_THz_ refers to the wavevector of incident THz pulse. E_THz_ and H_THz_ refer to the electrical components and magnetic components respectively. The lateral length of eSRR/CeSRR is L = 50 μm. The arm length of eSRR/CeSRR is l = 36 μm. The linewidth of the arm length of eSRR/CeSRR is w = 4 μm. The gap of eSRR/CeSRR is g = 2 μm. (**d**) The microscopic image of type-I and type-II self-complementary metamolecules. The zoom-in image shows the contact point < 1 μm^2^.

The THz frequency response of the MMs were measured in the frequency range from 0.2 THz to 1.2 THz by a fiber-coupled THz-TDS system using InGaAs/GaAlAs superlattice THz emitter and THz detector (TERA K15, Menlosystem GmbH). The Lock-In amplifier integrate the detected THz signal at the time constant of 100 ms. The diameter of THz focal area is 2 mm, which covers more than 10 unit cells of each meta-molecule since the lattice period of each MM is 100 μm. The temporal window is 15 ps in order to avoid the etalon echoes from the SI-GaAs substrate. The THz transmitted spectra in time domain is recorded under nitrogen atmosphere in order to avoid the absorption from steam vapour in air. The THz radiation is incident normally onto the surface of MMs. Meanwhile, another piece of 625 μm-thick SI-GaAs wafer is used as reference sample. Thus, the THz transmittance is extracted from Fourier transforms of the measured time-domain electric fields, which is defined as:

1 $$T(v)=|{E}_{sample}(v)/{E}_{ref}(v)|,$$

where E_sample_(v) and E_ref_(v) are the Fourier transformed electric fields through the sample and reference, respectively. T(v) is the transmittance as a function of THz frequency. Here, we address that the CC is naturally a kind of MM even though it does not contain eSRR/CeSRR. Therefore, the T_MM_(v) refers to the THz transmittance of two types of self-complementary MM, while T_c_(v) refers to the THz transmittance of CC itself shown in Fig. 2(a). Since the EIT effect originate from the destructive interference of eSRR and CeSRR, the intrinsic modes of these two types of resonators need to be excited simultaneously by incident THz. As such, we focus on the result of incident THz polarization perpendicular to the gap of eSRR and parallel to the inversed gap of CeSRR. There is no EIT phenomenon excited by another orthogonally polarized THz beam. (see Supplementary Fig. S1).

### Analysis

Since CC is a type of MM naturally, it has its own intrinsic mode. In order to exclude the influence of aforementioned intrinsic mode of CC, we propose an effective transmittance function Z(v), which get rid of the influence of intrinsic resonance of CC. It is the function of transmittance of self-complementary MM T_MM_(v) divided by the transmittance of CC Tc(v), shown as below:

2 $$Z(v)=|{T}_{MM}(v)/{T}_{c}(v)|,$$

The energy dissipation at the transparency windows of EIT effect can be derived from the Q factors^34^:

3 $$Q=\frac{{\nu }_{T}}{{\rm{\Delta }}{\nu }_{T}},$$

The linewidth of v_T_ is the full-width at half-maxima (FWHM) of the transparency window Δv_T_. In our experiment, the transparency windows are constructed by the two side-modes v_L_ and v_H_. Both modes are not at the same level in transmission spectrum. One is relatively swallower; the other one is relatively deeper. As such, we select the bottom of the transmittance minimum of the swallower side-mode as the base-line. The heights are from the maximum of the transparency window to this base-line. Then, the FWHM is the horizontal distance in between the spectral slopes of two side-modes at the half-height. The Δτ refers to the group delay of THz wave packet at the transparency window, which can be retrieved from the equation as below^28^:

4 $${\rm{\Delta }}\tau =-\,\frac{d\phi }{2\pi d\nu },$$

Here, φ and v refer to the effective phase and frequency of THz transmission spectrum, respectively. The effective phase can be calculated from the equation as below:

5 $$\phi ={\phi }_{T}-{\phi }_{ref}+kD.$$

Here, φ_T_ is the measured phase spectrum of our MMs, and φ_ref_ is the phase spectrum of reference; k is the wave-number of free space and D is the distance between input and output ports^35,36^. Since the phase of free-space is initially subtracted from the measured phase of MMs, an additional phase delay of free-space with the thickness of 625 μm was manually added to the subtraction.

### Simulation

THz transmittance (S parameters) as well as the surface currents and electromagnetic field distribution at resonance modes are simulated on the platform of CST Microwave Studio^TM^. The time-domain solver is adopted with the unit-cell boundary conditions in the x-y plane of one unit cell. The X-axis is set as electric boundary, while the Y-axis is set as magnetic boundary. The boundary condition along Z-axis is open. The input-and-output ports along the z direction are set 325 μm away from the front-side and back-side of MM respectively. In each port, only one mode is taken in account. In our simulation, we use hexahedral mesh of 9080000 cells. The mesh density is 40 lines per wavelength. The permittivity of SI-GaAs is ε_GaAs_ = 12.94 with a loss tangent of 0.006.

## Results

**Figure 3:**
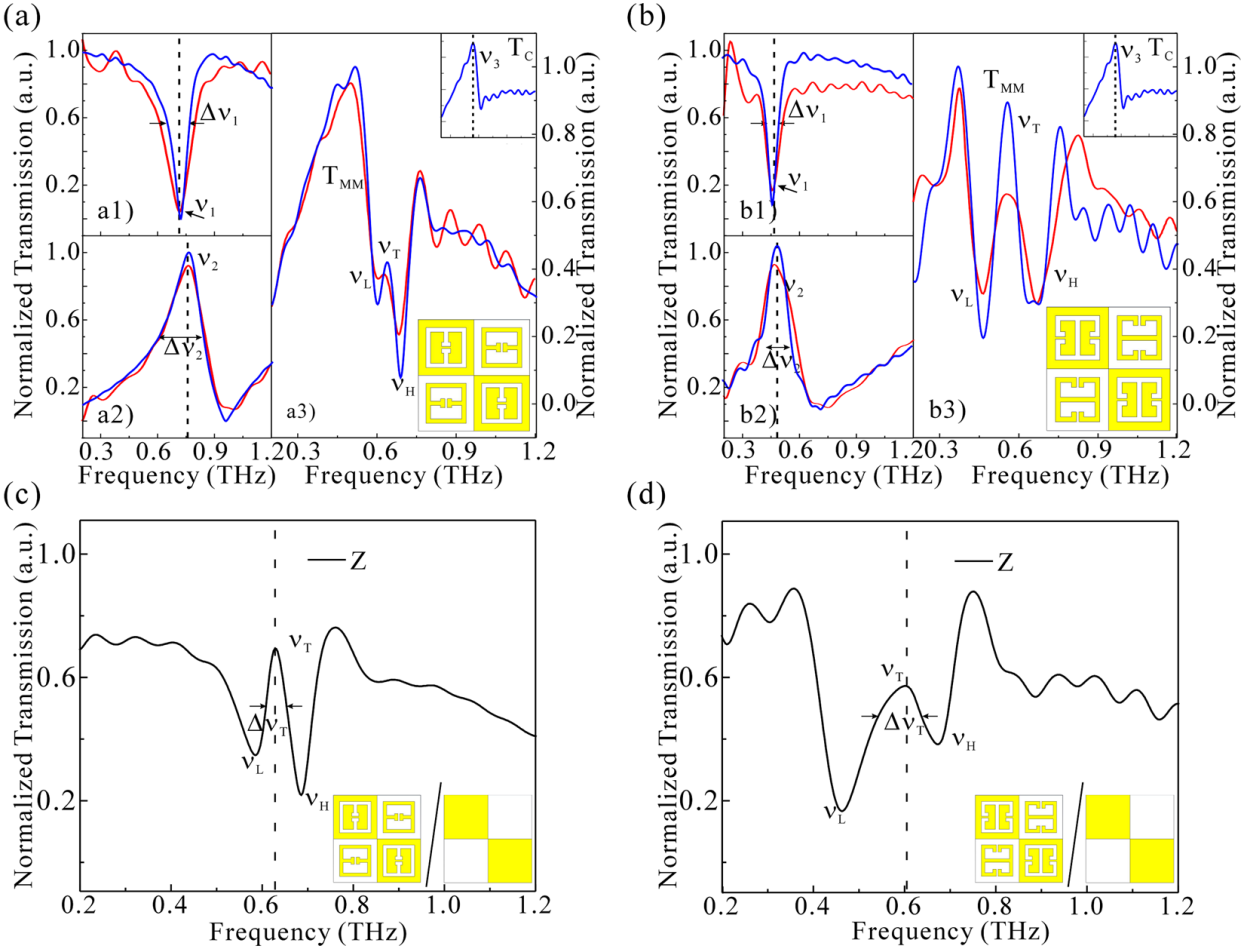
(**a**) The normalized THz transmittance of type-I self-complementary MMs. (**b**) The normalized THz transmittance of type-II self-complementary MMs. Blue solid-line: simulated THz transmittance. Red solid-line: measured THz transmittance. (**a**1) and (**b**1): resonance of eSRR; (**a**2) and (**b**2): resonance of CeSRR; T_MM_: THz transmittance of type-I and type-II self-complementary MM in (**a**3) and (**b**3); T_c_: THz transmittance of CC. Inset: the pattern of type-I and type-II self-complementary MM. The normalized function of Z, which is the value of T_MM_ divided by T_c_. The Z of (**c**) type-I and of (**d**) type-II self-complementary MMs, respectively.

The THz transmittance of sample is measured by THz-time domain spectroscopy (THz-TDS). The incident THz wavevector K_THz_ is along the normal line of the MM, as shown in Fig. 2(b,c). Thus, the incident THz polarization is perpendicular to the gap of eSRR but parallel to the inversed gap of CeSRR. Therefore, the two types of resonators will be excited simultaneously. In this case, there are no dark-resonators in our designed MM. Figure 2(a) shows the Croatian checkerboard pattern (CC), which consists of alternating metal squares and vacant squares. The metal squares connect one another at the corner. Such a pattern is like the escutcheon in the seal of Croatia. Therefore, each unit-cell of CC contains dual metal squares and vacant squares. Our self-complementary MM is based on a CC of 100 × 100 unit cell. A metal eSRR is in the center of vacant square area, and a complement (CeSRR) of the same size is in the center of the metal square area. The rectangular eSRR/CeSRR is of 36 μm length and 4 μm width with a 2 μm gap. The lattice period of eSRR/CeSRR is 50 μm. Since the THz response of eSRR/CeSRR relies on its gap position, two types of self-complementary MM are presented. One MM is of the central gap eSRR (type-I self-complementary MM) shown in Fig. 2(b), and the other one is of the arm-gap eSRR (type-II self-complementary MM) shown in Fig. 2(c). Therefore, the lattice period of both types of MM is 100 μm, as is twice to that of eSRR or CeSRR. The major parameters of type-I and type-II MMs are identical, such as period of unit cell, the length and width rectangular square, the gap size. The as-fabricated self-complementary MMs are shown in Fig. 2(d). The THz transmittances of the eSRR and CeSRR resonators are shown in Fig. 3(a1,a2)-(b1,b2), which is derived from the fast Fourier transform of the THz time domain data. A Lorenz line-shape resonance v_1_ occurs in THz spectrum when the THz polarization is perpendicular to the gap of eSRR. The resonance of CeSRR v_2_ is 50 GHz higher than the v1 to both types of MM. Owing to the metal losses and a high dielectric constant (ε_GaAs_ = 12.94) of 625 μm thick substrate, a 50 GHz frequency deviation occurs in between original eSRR and its inversed structure CeSRR. The CC is a kind of MM, and its THz response relies on the contact point as well as the geometric size of each piece of metal squares^33^. As shown in the inset of Fig. 3(a3)-(b3), a transmission maximum occurs in the THz spectrum termed as mode v_3_. This intrinsic mode of CC v_3_ is not Lorentzian line shape. In our case, the unit cell of CC connected each other so that the inductive coupling between the unit-cell of CC dominates the intrinsic mode v_3_. Owing to the broken contacts in between the metal square on the as-fabricated MM, the deviation between simulated and experimental THz transmittance become inevitable, as shown in Figs 3(a3)-2(b3). The spectral details of elementary resonators of two types of MM as well as the CC are listed in Table 1.

**Table 1:** The intrinsic mode frequencies of basic resonators.

Basic resonators	eSRR v1	CeSRR v2	CC v3
Type-I MM	0.71 THz	0.76 THz	0.57 THz
Type-II MM	0.46 THz	0.49 THz	0.57 THz

**Figure 4:**
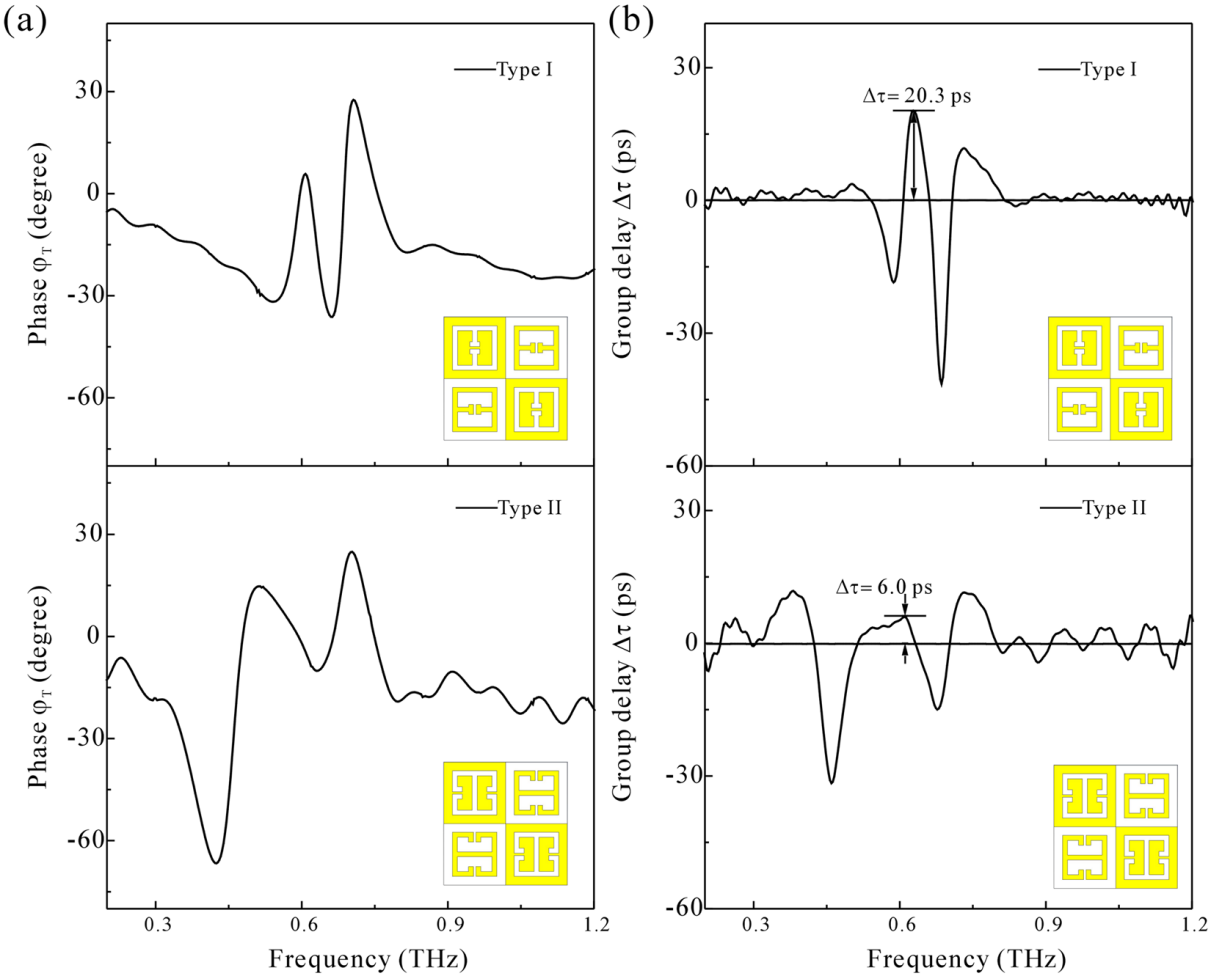
(**a**) The φ_T_ as a function of frequency of type-I and type-II self-complementary MM, correspondingly. (**b**) The Δτ as a function of frequency of type-I and type-II self-complementary MM, correspondingly.

Obviously, the relation of resonance modes in type-I pattern is v_3_ < v_1_ < v_2_; while that in type-II becomes v_1_ < v_2_ < v_3_. The THz response of two types of self-complementary MM are shown in the Fig. 3(a3)-(b3), a transparency window v_T_ appears to be in between the two transmittance minimum, one side-mode at low frequency component is termed as v_L_, and the other side-mode at high frequency component is termed as v_H_. The spectral profile below the side-mode v_L_ is a sharp increasing slope; while that beyond side-mode v_H_ is a slowly decreasing slope. Such a behavior is attributed to the spectral background of CC. With the help of equivalent transmission function Z(v), one can achieve a EIT effect on a relatively flat spectrum background, as are shown in Fig. 2(c,d). The parameters of transparency windows and side-modes are presented in Table 2. The transparency windows of type-I and type-II MMs are relatively close in frequency range, however, the width of window of type-I is only half to the that of type-II. Higher Q indicates a lower rate of energy loss relative to the stored energy of the resonator, where the oscillations die out more slowly. As such, the group velocity of propagating THz wave packet at transparency window of high Q factor is assumed to be slower than at transparency window of low Q factor. Figure 4(a) shows the phase spectrum φ_T_ of our MMs, where a phase transition is found obviously at the v_L_ and v_H_ modes. Following Eqs (4) and (5), a picoseconds-level group delay appears at transparency windows in MMs, as shown in Fig. 4(b). To the type-I MM, the Δτ achieves 20.3 ps. However, the Δτ of type-II MM is only 6 ps. The difference of THz slow light indicates that the origin of THz slow light at transparency window are different between type-I and type-II MM. Conventionally, the destructive interference of two basic resonators at the same resonance frequency leads to the EIT effect so that the transparency window should be identical to the intrinsic mode of basic resonator shown in Fig. 1. In our case, however, the transparency windows of both types of MMs (v_T_) are not identical to each one of the intrinsic modes eSRR (v_1_), CeSRR (v_2_), or CC (v_3_). Therefore, the directly destructive interference of eSRR and CeSRR resonators can be excluded from the mechanism of EIT effect in self-complementary MMs. Alternatively, one assumption of such a EIT effect is the hybridization of eSRR and CeSRR results in new resonance modes, which constructs a transparency window, just like the conductively coupled dimer and trimer MMs in previous works^35,36^. Therefore, one needs more evidence to reveal the origin of this EIT effect. With the help of numerical analysis of surface currents and electromagnetic field distribution, the origin of EIT is revealed.

**Table 2:** The v_L_, v_T_ and v_H_ of MMs of type-I and type-II MM.

	v_L_ (THz)	v_T_ (THz)	Δv_T_ (GHz)	Q	v_H_ (THz)
Type-I MM	0.6	0.63	43	14.7	0.69
Type-II MM	0.46	0.60	91	6.6	0.67

## Discussion

**Figure 5:**
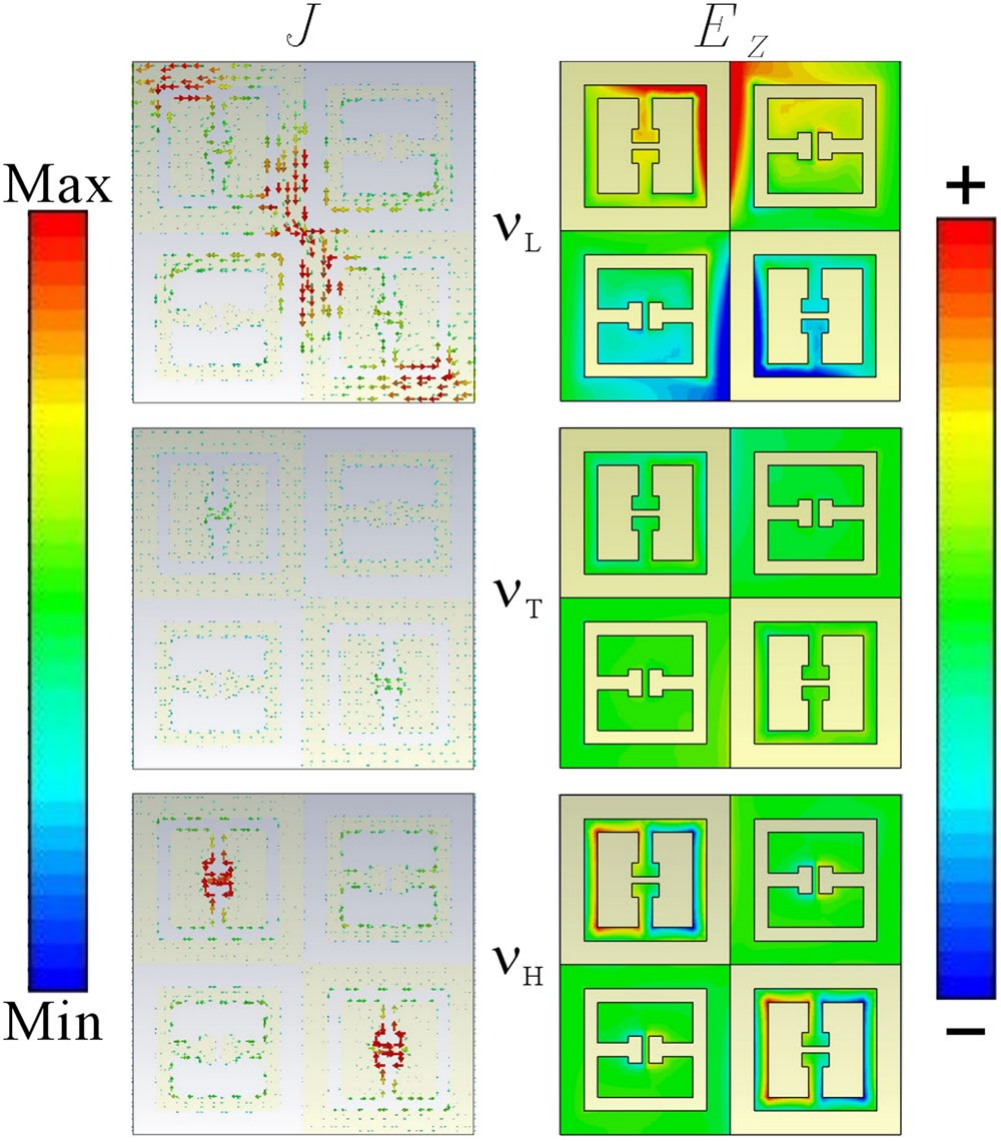
Electromagnetic energy distribution of type-I MM: J and E_z_ refer to the surface current and the electrical field along Z axis, correspondingly. The v_T_, v_L_ and v_H_ refer to the transparent window, low-frequency side-mode and high frequency side-mode respectively. The color bar refers to the relative strength of surface currents and electrical field.

The surface currents as well as the electrical field distribution of v_L_, v_T_, and v_H_ mode of type-I self-complementary MM are shown respectively in Fig. 5. The induced surface current as well as the electrical field achieves minimum at the transparency window v_T_ mode. Interestingly, the surface currents on the CeSRR dominate the side-mode v_L_ and v_H_. The contribution of eSRR almost can be neglected. Especially, the v_L_ side-mode experiences a strong conductive coupling in between the adjacent CeSRRs via the contact point. The currents leaks from the right-down corner of the left-upper CeSRR to the left-upper corner of the right-down CeSRR. Part of the current flows along the outer-edge of CeSRR from upside to the downside, and part of the currents flows from left to right. Such a behavior make the connected CeSRR become a twisted dipole from left-upper to right-down, which can be confirmed from mapping the electrical field distribution. Therefore, the LC mode can be excluded in the origin of side-mode vL. Meanwhile, the side-mode v_H_ is dominated by the coupled local LC loop at the vicinity of the inversed gap area of CeSRR. Upon to the previous works on the eSRR and CeSRR, the LC loop flows along the whole metal edge of the resonators^2^. In our case, however, the route of LC loop on CeSRR is much smaller than the intrinsic mode of CeSRR, which make its frequency different from the intrinsic mode of CeSRR, shown in Fig. 3. Interestingly, there is almost no currents leakage in between adjacent CeSRR via contact point at this frequency. Therefore, v_H_ side-mode attribute to the near-field coupling of CeSRR, which make the localized LC mode resonating in phase. Herein, The transparency window v_T_ mode is constructed by the side-mode v_L_ and v_H_, where the LC mode on eSRR does not contributes to the generation of EIT.

**Figure 6:**
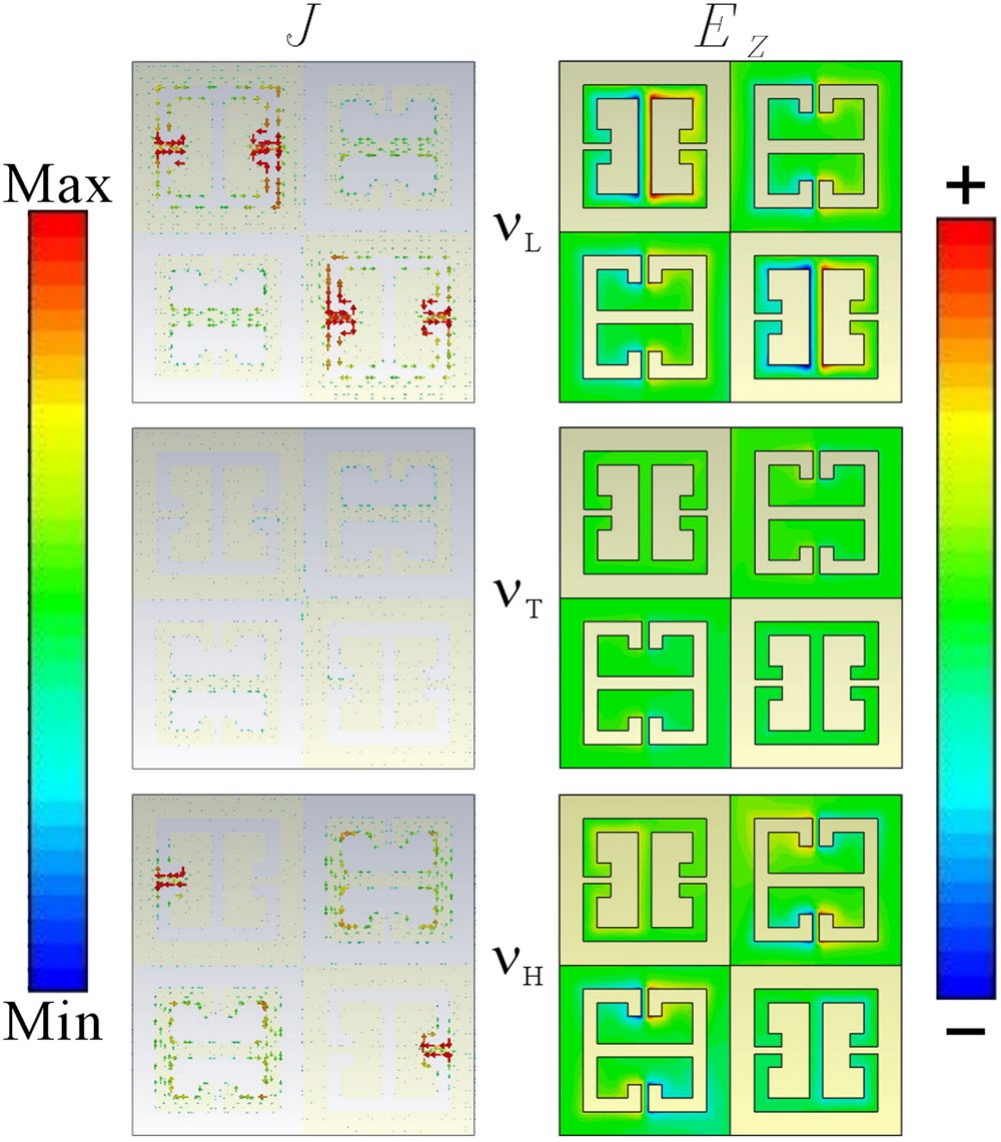
Electromagnetic energy distribution of type-II MM: J and Ez refer to the surface current and the electrical field along Z axis, correspondingly. The v_T_, v_L_ and v_H_ refer to the transparent window, low-frequency side-mode and high frequency side-mode respectively. The color bar refers to the relative strength of surface currents and electrical field.

The origin of v_L_, v_T_, and v_H_ mode of type-II self-complementary MM is different from the type-I, as shown in Fig. 6. The surface current as well as the electrical field achieves minimum at the transparency window vT mode as well. To the side-mode vL, however, the incident THz wave excited multiple localized LC resonance in the two CeSRRs of MM. In agreement to the type-I MM, such a LC loop flows at the vicinity of the inversed gap area of CeSRR. Since the two inversed gaps are in mirror symmetry, the current flows clockwise at one side but counter-clockwise at the other side. Such a current flow leads to the electrical field distribution in mirror symmetry correspondingly. Meanwhile there is not obvious current leakage in between the adjacent CeSRR via contact point. Compared to the currents on CeSRR, the LC loop on eSRR is relatively weaker so that the contribution of eSRR can be neglected as well. To the side-mode v_H_, the current loop appears to be on both CeSRR and eSRR. A couple of LC loop in up-and-down mirror symmetry is found along the metal inner-edges of the two eSRRs. Meanwhile, a couple of parallel currents occur at the vicinity of the inversed gap area of CeSRR, which make the CeSRR become an equivalent dipoles. The current direction is parallel to the electrical component of the incident THz wave E_x_. Thus, the metal area of inversed gap plays the role as a cut-wire so as to support the dipole oscillation. Therefore, hybridization occurs between the localized LC resonance of eSRR and the dipole oscillation on the metal CeSRR, which dominate the side-mode v_H_. These behavior are very much like the EIT effect in conductively couple dimer and trimer MMs, where the new-generated side-modes construct a transparency windows rather than the destructive interference in between the intrinsic modes of basic resonators^35,36^.

## Conclusion

In summary, an EIT phenomenon at THz frequency band is observed in self-complementary MMs in Croatian checkerboard. The MMs contain a couple of eSRR and its inversed structure CeSRR. For the type-I MM, the each eSRR and CeSRR has only one gap. For the type-II MM, there are two gaps on each eSRR and CeSRR. A transparency windows appears at 0.63 THz (type-I MM) and 0.6 THz (type-II MM), which are different from the intrinsic modes of basic resonators correspondingly. The Q factors of transparency windows indicate that type-I MM has a higher energy storage-to-loss ratio than type-II MM at slow light channel. To the type-I MM, its maximum slow light achieve 20.3 ps; while that of type-II MM is only 6 ps. To the type-I MM, the surface currents indicate that the only the CeSRR contribute to side-modes of type-I MM. The low-frequency side-mode attributes to the current leakage between the conductively couple CeSRR via contact point. The high-frequency side-mode originates from the localized LC resonance. To the type-II MM, however, the surface currents and electric energy distribution reveal a LC resonance on CeSRR dominates the low-frequency side-mode; while a couple of parallel dipole oscillation on metal structure of CeSRR hybrids with LC resonance on eSRR, which gives arise to the high frequency side-modes. The different side-modes construct the transparency window of EIT, which leads to different THz slow light channels.

## Supplementary information

Dataset 1
