# Supplementary material for: Terahertz electromagnetically-induced transparency of self-complementary meta-molecules on Croatian checkerboard
Source: Sci Rep. 2019 Apr 17;9:6205. doi: 10.1038/s41598-019-42038-8 (PMC6470151; doi:10.1038/s41598-019-42038-8)
Supplement: Supplementary file 1 — Dataset 1 [file 41598_2019_42038_MOESM1_ESM.pdf]

Supplemental Online Material

Terahertz electromagnetically-induced transparency of self-complementary meta-molecules on Croatian checkerboard

ZHENYU ZHAO[1)], XIAOBO ZHENG, WEI PENG, JIANBING ZHANG,

HONGWEI ZHAO, AND WANGZHOU SHI1

In this supplemental online material, we present the measured data of THz transmittance of self-complementary metamolecules on Croatian checkerboard at the incident THz polarization parallel to the metal gap. The figures of the main article and of this supplemental online material are referred to as "Fig. S1", respectively.

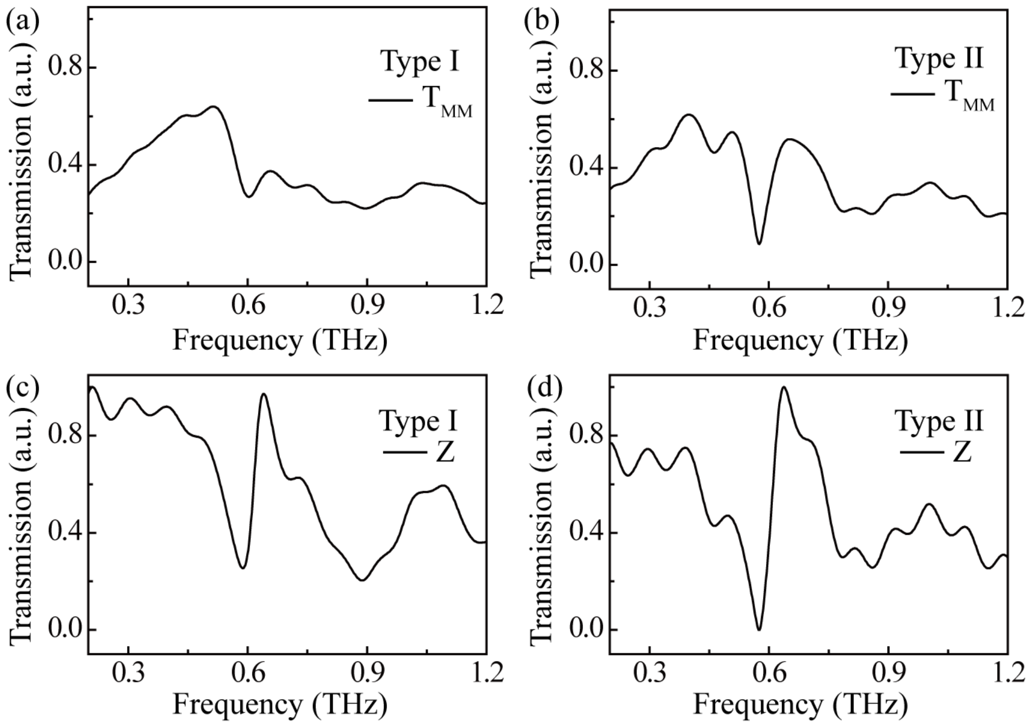

Fig. S1. (a) The simulated THz transmittance of type-I self-complementary MMs. (b) The simulated THz transmittance of type-II self-complementary MMs. The Z of (c) type-I and of (d) type-II self-complementary MMs, respectively. The incident THz polarization is along the X-axis.

[1)] Electronic mail: zyzhao@shnu.edu.cn
